# The Neuroimmune Response to Surgery – An Exploratory Study of Trauma-Induced Changes in Innate Immunity and Heart Rate Variability

**DOI:** 10.3389/fimmu.2022.911744

**Published:** 2022-07-07

**Authors:** Malin Hildenborg, Jessica Kåhlin, Fredrik Granath, Anna Schening, Anna Granström, Anette Ebberyd, Lena Klevenvall, Henrik Zetterberg, Jinming Han, Todd T. Schlegel, Robert Harris, Helena Erlandsson Harris, Lars I. Eriksson

**Affiliations:** ^1^ Department of Physiology and Pharmacology, Section of Anesthesiology and Intensive Care Medicine, Karolinska Institutet, Stockholm, Sweden; ^2^ Function Perioperative Medicine and Intensive Care, Karolinska University Hospital, Stockholm, Sweden; ^3^ Clinical Epidemiology, Department of Medicine, Karolinska Institutet, Stockholm, Sweden; ^4^ Center for Molecular Medicine, Department for Medicine Solna, Karolinska Institutet, Stockhlom, Sweden; ^5^ Rheumatology Unit, Center for Molecular Medicine, Department for Medicine Solna, Karolinska Institutet, Stockhlom, Sweden; ^6^ Department of Psychiatry and Neurochemistry, Institute of Neuroscience and Physiology, Sahlgrenska Academy at University of Gothenburg, Mölndal, Sweden; ^7^ Clinical Neurochemistry Laboratory, Sahlgrenska University Hospital of Gothenburg, Mölndal, Sweden; ^8^ Department of Molecular Neuroscience, University College London Institute of Neurology, London, United Kingdom; ^9^ Hong Kong Center for Neurodegenerative Diseases, Hong Kong SAR, China; ^10^ Department of Clinical Neuroscience, Karolinska Institutet, Centre for Molecular Medicine, Karolinska University Hospital, Stockholm, Sweden; ^11^ Department of Molecular Medicine and Surgery, Karolinska Institutet, Stockholm, Sweden; ^12^ Nicollier-Schlegel SARL, Trélex, Switzerland

**Keywords:** surgery, innate immunity, heart rate variability (HRV), inflammation, neuroimmune alterations, perioperative neurocognitive disorders (PND)

## Abstract

Surgery triggers a systemic inflammatory response that ultimately impacts the brain and associates with long-term cognitive impairment. Adequate regulation of this immune surge is pivotal for a successful surgical recovery. We explored the temporal immune response in a surgical cohort and its associations with neuroimmune regulatory pathways and cognition, in keeping with the growing body of evidence pointing towards the brain as a regulator of peripheral inflammation. Brain-to-immune communication acts through cellular, humoral and neural pathways. In this context, the vagal nerve and the cholinergic anti-inflammatory pathway (CAP) have been shown to modify peripheral immune cell activity in both acute and chronic inflammatory conditions. However, the relevance of neuroimmune regulatory mechanisms following a surgical trauma is not yet elucidated. Twenty-five male patients undergoing elective laparoscopic abdominal surgery were included in this observational prospective study. Serial blood samples with extensive immune characterization, assessments of heart rate variability (HRV) and cognitive tests were performed before surgery and continuing up to 6 months post-surgery. Temporal immune responses revealed biphasic reaction patterns with most pronounced changes at 5 hours after skin incision and 14 days following surgery. Estimations of cardiac vagal nerve activity through HRV recordings revealed great individual variations depending on the pre-operative HRV baseline. A principal component analysis displayed distinct differences in systemic inflammatory biomarker trajectories primarily based on pre-operative HRV, with potiential consequences for long-term surgical outcomes. In conclusion, individual pre-operative HRV generates differential response patterns that associate with distinct inflammatory trajectories following surgery. Long-term surgical outcomes need to be examined further in larger studies with mixed gender cohorts.

## 1 Introduction

The inflammatory response evoked by surgery rapidly spreads to remote organs *via* a temporal cascade of molecular and cellular signaling pathways within the innate immune system. Orchestration of this trauma-induced immune activation is dependent on molecular and neural regulatory pathways that result in a multiphasic response, including pro- and anti-inflammatory as well as resolving processes (1–3).

There is a growing body of evidence supporting an important role of the brain in regulation of acute and chronic inflammation through several molecular and cellular mechanisms (4–6). The cholinergic anti-inflammatory pathway (CAP) involves the vagal nerve in bidirectional brain-to-immune communication. In brief, in-bound afferent vagal-nerve signaling from the periphery provides information about innate immune activity and systemic inflammation, while the out-bound efferent vagal nerve activity provides counter-balancing regulatory properties targeting systemic immune cells (7, 8). This latter effect is achieved *via* vagal and splenic nerve-mediated adrenergic and cholinergic transmission in the spleen and other lymphoid tissues, ultimately promoting JAK-STAT3 and NFκB-dependent downregulation of immune signaling within blood-borne or resident macrophages (9–14). The afferent and efferent limbs of this neural route thus form a neural regulatory reflex pathway through which the brain communicates with the peripheral immune system and can modulate systemic inflammation.

Cardiac vagal nerve activity can be readily approximated by monitoring heart rate variability (HRV) (15–17). There is a close association between HRV and systemic inflammation, such that changes in HRV patterns can be used to detect prodromal states of acute illness in patients with systemic inflammation, as reported for severe infection or sepsis (18–23). In addition, in patients with chronic inflammatory disorders such as rheumatoid arthritis (RA) and inflammatory bowel disease (IBD), vagal nerve stimulation dampens systemic inflammation and promotes inflammatory resolution with objective and subjective clinical improvement (24, 25). Vagal nerve signaling can thus be used for inflammatory sensing and monitoring, as well as for inflammatory interventions.

Recent observations in surgical patients suggest that surgery-induced immune activation with impaired inflammatory resolution might lead to long-term postoperative impact, especially long-term deficits on higher brain functions including neurocognition (26–28). However, we lack an understanding of the role of the brain and the autonomic nervous system during peripheral immune response to surgery and related long-term post-operative outcomes.

The primary purpose of this study was to explore surgery-induced temporal changes in vagal nerve activity and its association with systemic innate immune molecular and cellular activities. The secondary aim was to investigate whether different vagal nerve response patterns are linked to long-term postoperative neurocognitive outcomes.

## 2 Methods

### 2.1 Subjects

This observational prospective study was approved by the Stockholm Regional Ethical Review board (2016/1745-31/1), registered at ClinicalTrials.gov (NCT03055325) and conducted in accordance with the Declaration of Helsinki 2013.

Twenty-five patients, aged 45-75 years, scheduled for elective robot-assisted laparoscopic prostatectomy (RALP) were included after informed consent. Patients were defined as ASA I-II (American Society of Anesthesiologists) and pre-operative cognitive capacity was determined using Mini Mental State Exam (MMSE). Data collection took place between January 2017 and October 2019 at the Karolinska University Hospital Stockholm, Sweden.

Exclusion criteria include neurodegenerative diseases, significant psychiatric illness or a MMSE score of ≤ 23, previous stroke, cardiac illnesses including active arrhythmia, chronic pain or inflammatory disease such as RA or IBD, medication with steroids, statins, ß-blockers or anti-cholinergic drugs, diabetes mellitus or any other condition known to cause autonomic dysfunction, substance abuse and previous splenectomy. Also, surgery within 6 months, cancer treatment within 12 months or infectious disease treatment the previous month and presumed uncooperativeness or legal incapacity were grounds for exclusion.

### 2.2 Anesthesia, Surgical and Postoperative Care

Patients’ medical history was assessed at inclusion. Complementary and perioperative information was extracted from the medical record system (TakeCare) at Karolinska University Hospital.

No routine premedication was administered. Prior to surgery, patients received intrathecal bupivacaine (10 mg) and sufentanil (5 μg) followed by induction of general anesthesia using remifentanil, propofol and rocuronium. Anesthesia was maintained using desflurane (0.7 – 1.0 MAC) and a continuous IV infusion of remifentanil (B. Braun Perfusor^®^ Space). All patients received a radial arterial catheter for invasive arterial blood pressure monitoring and serial blood sampling. The laparoscopic robotic surgical procedure was performed in the Trendelenburg position (30-45 degrees) with intra-peritoneal insufflation of CO_2_ (12 mmHg). After completion of surgery patients were transferred to the post-anesthetic care unit (PACU). Intravenous ketobemidone combined with acetaminophen was administered before emergence from general anesthesia and repeated intermittently as needed to achieve analgesia. All patients were prescribed oral oxycodone and a 7-day treatment with subcutaneous low molecular weight heparins (LMWHs) following hospital discharge.

### 2.3 Study Protocol

**Figure 1:**
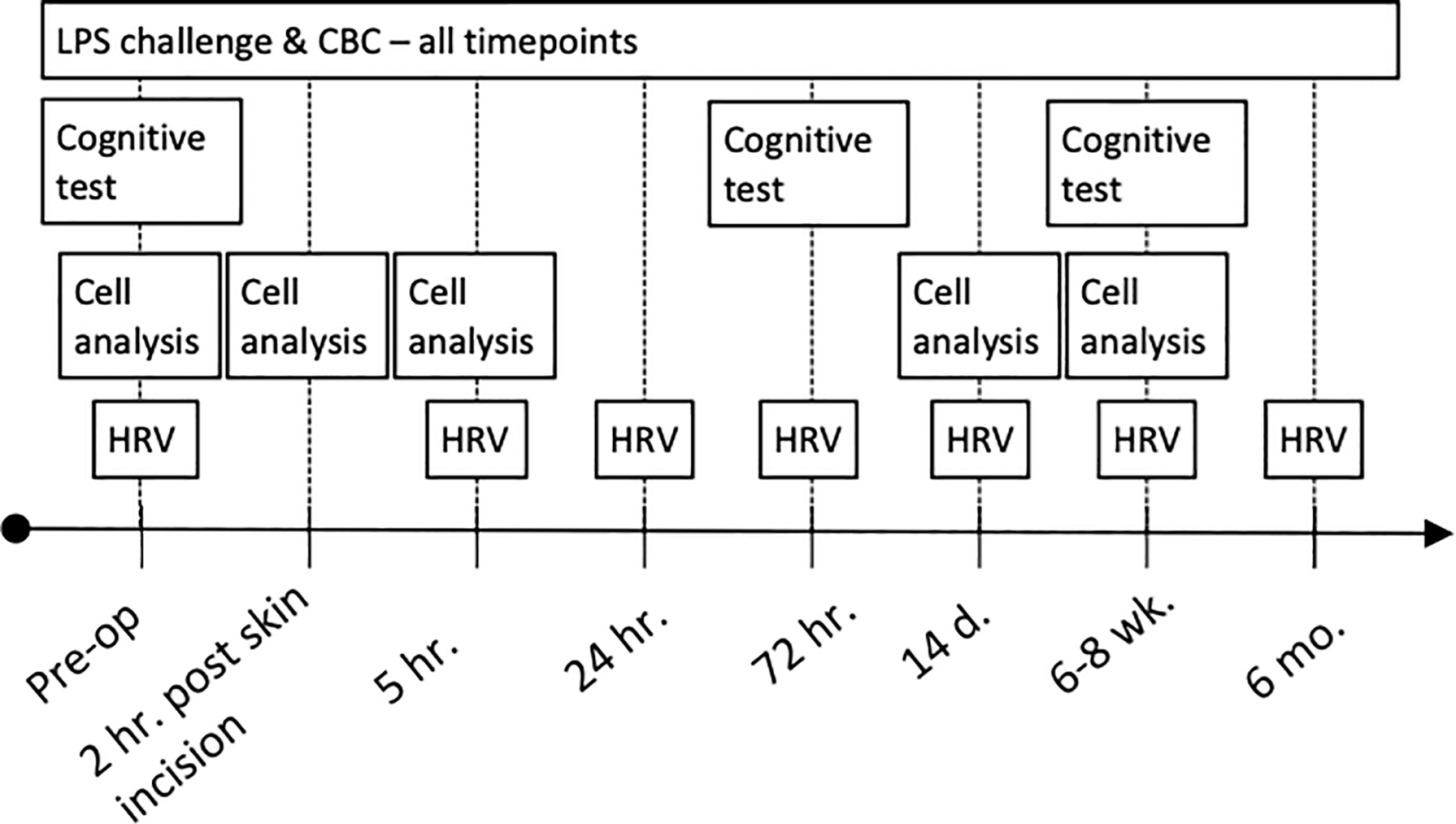
Clinical trial profile. CBC, complete blood count; LPS, lipopolysaccharide.

Serial blood sampling, HRV-recordings and cognitive tests were performed starting before surgery and continuing for up to 6 months after surgery as outlined in **Figure 1**.

### 2.4 HRV and QTV

Five-minute 12-lead ECG recordings to achieve a minimum of 256 beats were acquired using a computer-assisted ECG device (Advanced ECG, Space EKG Technology, Trélex, Switzerland and Cardiax software IMED Kft. Budapest, Hungary). Recordings were made in the supine position, in the morning (except for the recording 5 hours post-skin incision), during fasting conditions with patients resting for a minimum of 5 minutes before the start of recordings, and with numerical response scale (NRS) score for pain <5.

**Figure 2:**
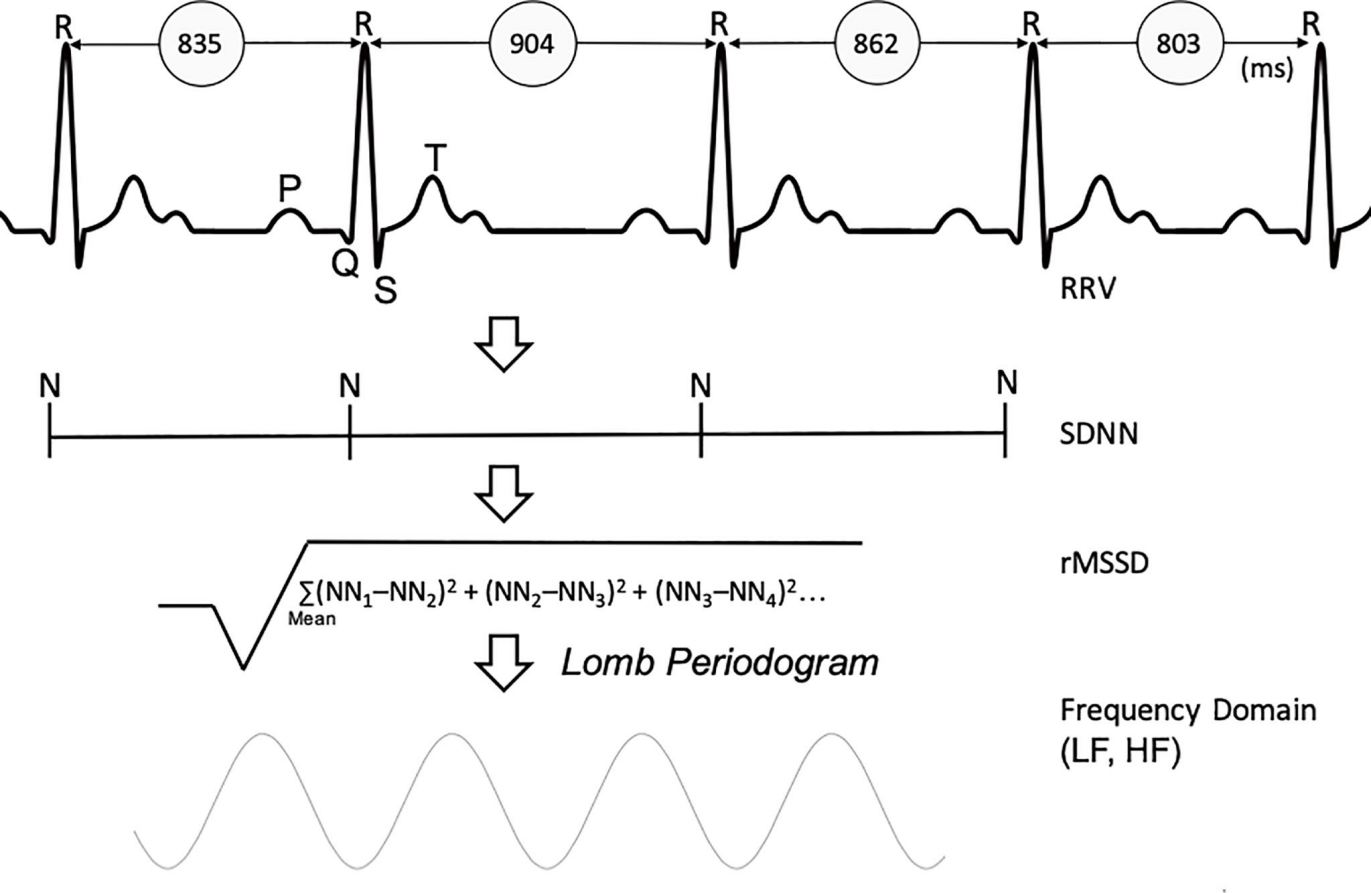
Derivation of Heart Rate Variability parameters. RRV, R-to-R variability; SDNN, standard deviation of normal-normal heart beats; rMSSD, root mean square of successive differences; LF, low frequency; HF, high frequency.

Time series for the RR and QT intervals were analyzed according to the Task Force of the European Society of Cardiology standards (16, 29). Specifically, analyses in the time domain included the standard deviation of normal-to-normal RR intervals (SDNN), the root mean square of the successive interval difference of normal-to-normal RR (rMSSD) and the first standard deviation (SD1) from the 256-beat Poincaré plot. In the frequency domain, the very low (VLF, 0.0–0.04 Hz), low (LF, 0.04–0.15 Hz), high (HF, 0.15– 0.40 Hz), and total (TP, 0.0–0.40 Hz) frequency powers of RR interval variability in natural log-transformed units (ln ms^2^/Hz) were calculated using the Lomb periodogram method (**Figure 2**) (30).

For QT interval variability (QTV), the QT variability index (QTVI) was calculated for the entirety of each ~5-min recording by utilizing the signals from lead II, specifically after the method of Starc and Schlegel (29, 31, 32). In patients without heart failure, supine resting (non-stressed baseline) QTV is believed to mostly reflect cardiac vagal activity (33).

### 2.5 Whole Blood Analyses

On each study occasion blood was sampled and immediately processed for *ex vivo* whole blood LPS stimulation. We also centrifuged and stored plasma and serum (-80°C) for later analyses. Furthermore, whole blood was processed for analysis of peripheral blood mononuclear cells (PBMCs) and by certified laboratories for T-cells and complete blood count (CBC).

#### 2.5.1 *Ex Vivo* Lipopolysaccharide Stimulation

One-hundred µL of whole blood (arterial or venous) was added to round-bottomed 96 well plates (Nunc) containing 96µL Dulbecco’s Modified Eagle Medium (DMEM), within 30 minutes from each sampling. Four µL of either LPS (*E.Coli* 0111:B4, Sigma, L2630, 0.5µg/mL) or phosphate buffered saline (PBS) alone was added to triplicate wells, rendering a final concentration of LPS of 10 ng/ml per well, where added. Blood cultures were incubated at 37°C, 5% CO_2_ for 4 hours on a rocking board. Three mM ATP (Sigma, A2383) was added for the last hour. Plates were then centrifuged for 10 min at 2000g and supernatants subsequently transferred to vials and stored at -20°C until assayed for concentrations of relevant inflammatory biomarkers content by Enzyme-linked immunosorbent assay (ELISA) techniques, according to the manufacturer instructions (R&D systems). OD values were recorded using a plate reader and SoftMax software.

#### 2.5.2 Cytokine Assessment

Serum biomarkers of systemic inflammation were analyzed using a high-throughput, multiplex immunoassay (Proseek^©^ Multiplex Inflammation) by PEA technology (Olink Proteomics AB, Uppsala, Sweden). Analysis was conducted using one sample (patient) per well. For each of 92 selected inflammatory proteins, two separate oligonucleotide-marked antibodies were applied. After binding by the antibody pair to its target, DNA polymerization provides a protein-specific reporter DNA-sequence for each detected protein molecule. The reporter DNA strands were then quantified using qPCR. The acquired Cq values were normalized and converted into Normalized Protein Expression Units (NPX), expressed on a Log2 scale.

ELISA was used for high-sensitivity C-reactive protein (hsCRP, Hycult Biotech, Cat no:HK369) and high mobility group box protein 1 (HMGB1) concentration was measured using a commercial kit (Tecan/IBL, Cat no: ST51011, Lot no: EHMG147).

#### 2.5.3 Cellular Analyses

CBC and T-cell analyses were performed by Karolinska University Laboratory, Stockholm, Sweden, using Sysmex XN-9000 for CBC processing. T-cell analysis for CD4+ and CD8+ expression was performed using an Aquios CL (Beckman coulter) which utilizes a direct volumetric single‐platform method with incorporated sample preparation with a monoclonal antibody mixture (anti‐CD45‐FITC [clone B3821F4A], anti‐CD4‐RDI [clone SFCI12T4D11], anti CD8‐ECD [SFCI21thyD3], anti‐CD3‐PC5 [clone UCHT1]) Beckman Coulter.

For PBMC isolation, whole blood was sampled using BD Vacutainer^®^ CPT™ Mononuclear Cell Preparation Tubes and processed within 3 hours of collection. PBMCs were then isolated according to the standard procedure (centrifuged at 1500 g for 20 min at room temperature) and washed with cold PBS (440 g for 10 min at 4°C). Single cell suspensions were plated in 96-well V-bottomed plates and stained for 20 min at 4°C. The cells were incubated with Alexa Fluor647 anti-human CX3CR1 (clone: 2A9-1, BioLegend), PerCP/Cy5.5 anti-human CD192 (CCR2) (clone: K036C2, BioLegend), APC/Cy7 anti-human CD68 (clone: Y1/82A, BioLegend), PE/Cy7 anti-human CD11b (clone: ICRF44, BioLegend), Alexa Fluor488 anti-human CD16 (clone: 3G8, BioLegend) and PE anti-human CD14 (clone: 63D3, BioLegend). Cells were acquired using a Gallios flow cytometer (Beckman Coulter) and analyzed using Kaluza software (Beckman Coulter).

### 2.6 Serial Cognitive Testing and Test Battery

Cognitive capacity was assessed using the International Study of Postoperative Cognitive Dysfunction (ISPOCD) test battery (34). The test was conducted at three time points by one of three trained investigators. The test battery consists of four parts, rendering seven variables for analysis. The visual verbal learning test (VLT) tests word recall in 3 trials and 1 delayed recall; the concept shifting test (CST) measures time (s) and errors in part C of the trial; the Stroop color word test (SCWT) measures time (s) and error in the third part of the test and finally the number of correct answers were recorded in the letter digit coding test (LDC).

Individual test results were compared to baseline (prior to surgery) rendering Z-scores for each test and a composite Z-score for overall performance. Results were further adjusted to age-matched controls to account for variability and practice effects (34). The 30-minute test was altered at each temporal assessment and conducted in a silent room. We defined poor cognitive performance as either a composite Z-score of >1.0 or as a Z-score of >2.0 in a single part of the test battery.

### 2.7 Statistical Analysis

Data are presented as mean value ± standard deviations (SDs) unless otherwise specified. Differences in HRV, cell and cytokine levels over time or between vagal subgroups were analyzed using repeated measures ANOVA and mixed-effects model with Bonferroni’s or Tukey’s tests for multiple comparisons. A principal component analysis (PCA) was applied to analyze systemic inflammatory and immune biomarkers to reduce dimensionality and thereby reduce the problem of mass-significance. The PCA was performed on the basis of the pre-operative measurements, and subsequently the three first principal components were identified. These construct variables are three differently weighted averages of the standardized measurements (i.e. having zero mean and unit variance) of the included biomarkers. The pre-operatively obtained weighting schemes were then applied to the standardized post-operative measurements at each timepoint. The obtained individual time trajectories of these construct variables (PC1-PC3) were compared between HRV-groups by mixed effects model analyses. The significance levels for the three time-group interaction tests obtained were Bonferroni corrected. An observed significant group-time interaction indicates that the latent pre-operative biomarker pattern identified by PCA is differentially affected by the surgical trauma in the HRV-groups. Furthermore, when a significant interaction was identified, analyses were performed on the subset of individual biomarkers with the largest weights in the corresponding construct variable (i.e >0.1 for PC2). Differences were considered significant if p<0.05. We utilized software GraphPad Prism version 8.00 software for Mac (GraphPad Software, La Jolla, CA, USA) and SAS version 9.4 (SAS Institute Inc., Cary, NC, USA).

## 3 Results

Of the twenty-five patients included, twenty-four had complete data sets and one patient was lost during follow up. Demographic and perioperative information is presented in **Table 1**.

**Table 1:** Patient characteristics and perioperative data.

		patients n = 25
Age, years	59 (45, 75)	
Sex, male, n (%)	25 (100)	
Weight, kg	84 (65, 97)	
Height, cm	181 (164, 194)	
Body Mass Index, kg/m2	25 (21, 30)	
Comorbidities Hypertension, n (%) Diabetes, n Nicotine use, n (%)	2 (8) 0 4 (16)	
ASA classification		
I, n; II, n; III & IV, n	14; 11; 0	
Mini Mental State Exam, score	30 (27, 30)	
Pre-operative laboratory results		
Blood hemoglobin, g/L	146 (133, 168)	
Serum creatinine, µmol/L	87 (66, 109)	
WBC count, x10^9/L	5,5 (3,7, 8,3)	
Ongoing medication		
Angiotensin converting enzyme inhibitor, n	2	
Opioids, n	1	
Educational level, years in school		
<9 yrs, n (%), high school	1 (4)	
9-12 yrs, n (%), gymnasium/college	10 (40)	
>12 yrs, n (%), higher education	14 (56)	
**Perioperative data**		
SPA, n (%)	25 (100)	
Propofol induction, mg	180 (70, 350)	n=24
Remifentanil, total amount, mg	3,14 (0,25, 5,02)	n=13
Rocuronium, mg	50 (40, 60)	n=18
Vasopressor, n	24	
Phenylephrine, tot amount, mg	0,2 (0,05, 0,65)	n=13
Ephedrine, tot amount, mg	27,5 (5, 45)	n=21
Duration of surgery, min	156 (76, 212)	
Bleeding, ml	100 (0, 400)	
Intravenous fluids, Acetated Ringer, ml	1650 (500, 2500)	n=22
Albumin, ml	250 (100, 250)	n=7
**Post-operative data**		
PACU length of stay, minutes	239 (145, 455)	
Hospitalization total, hours	32,2 (30,6, 58,1)	
Intravenous fluids, 24 hrs including OR, ml	2300 (1200, 3600)	
Medications		
IV opioid, mg oral morphine equivalents PlPACU to 24 hrs post-surgery	17 (7,5, 35)	
NSAID, n	1	
Clonidine (α-receptor antagonist), n	1	
Benzodiazepine, single dose pre-op, n	2	
Benzodiazepine, single dose post-op, n	1	
Droperidol, n	1	
**Post-operative NRS score**	Pain	Nausea
5 h	3 (0, 4)	0 (0, 4)
24 h	3 (0, 5)	0 (0, 6)
72 h	1 (0, 5)	0 (0, 3)
14 d	0 (0, 3)	0 (0, 0)
6-8 w	0 (0, 4)	0 (0, 0)
6 months	0 (0, 3)	0 (0, 0)

Values are median (min, max). Age in years. SPA, spinal anaesthesia; NMBA, neuro muscular blocking agents; PACU, Post-Anaesthesia Care Unit; NRS, Numeric Rating Scale; OR, operating room.

### 3.1 HRV and QTV

**Figure 3:**
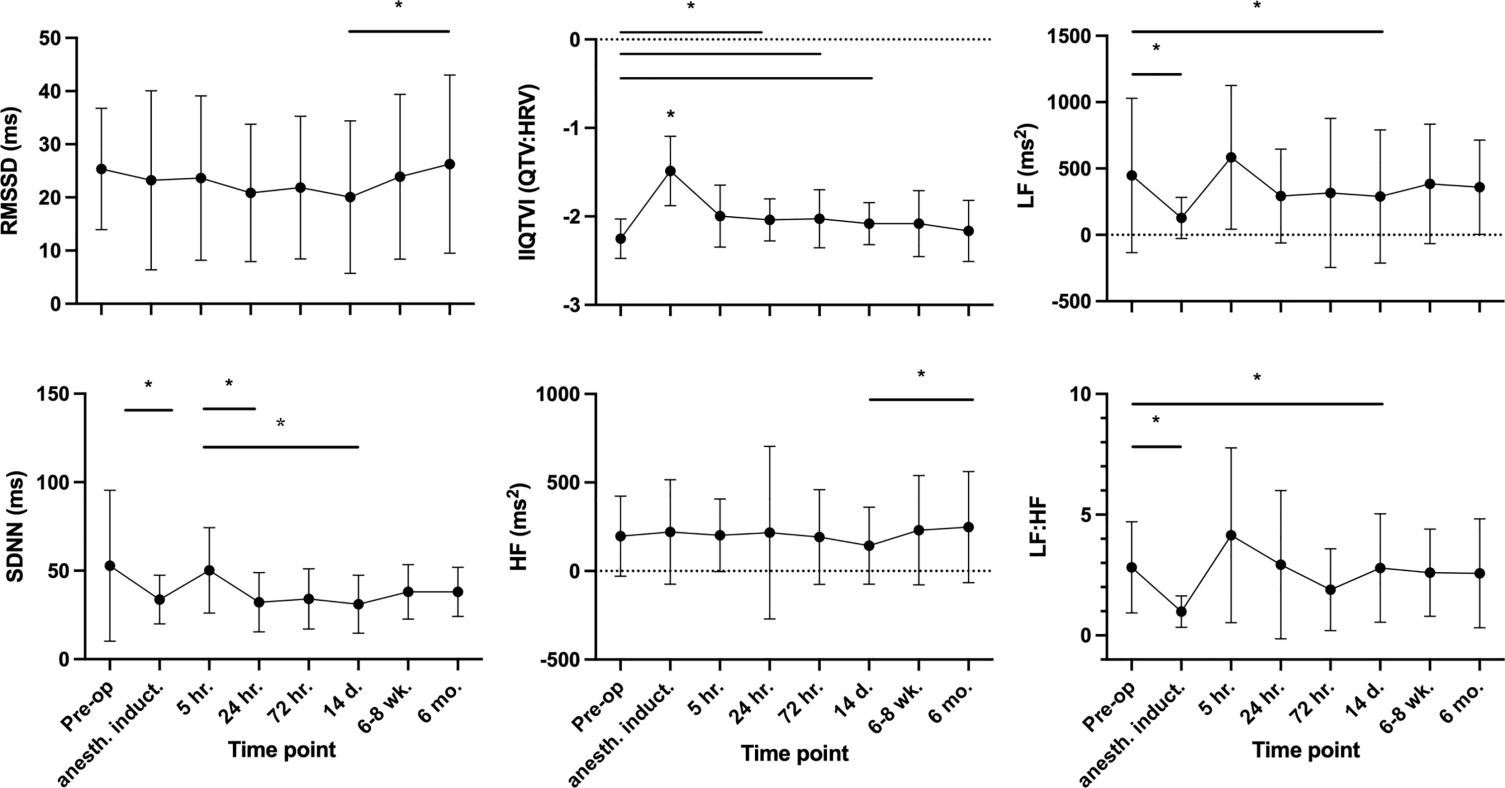
Temporal HRV trajectories. Mean values, SD. For the indexed parameter IIQTVI and the ratio LF : HF, a greater value implies less variability. * P-values ≤0.05, Tukey´s multiple comparison test.

The six HRV domains (rMSSD, SD1, HF, LF, LF:HF and SDNN) and IIQTVI were first characterized by exploring their reciprocal associations. Notably, SDNN, rMSSD, SD1 and HF all strongly correlated (R 0.9-1.0), as expected, while LF and especially IIQTVI changed in a distinctly different pattern (see **Supplementary Figure 1**, for coefficients). The pre-operative correlations were consistent over time. During anesthesia, HRV declined significantly, with SDNN -36% and LF -72% versus their respective baselines, and with IIQTVI increasing by 34%. At 5 hours after skin incision, HRV values had recovered from anesthesia (**Figure 3**). At an individual level, however, patients with a higher pre-operative variability displayed higher variability throughout the study period compared to patients with less dynamic variability, the 20% of patients with highest pre-operative variability in RMSSD having temporal coefficients of variation (CV) of 32-102% whereas the lowest 20% had CVs of 10-35%.

#### 3.1.1 HRV Phenotypes

Based on the preoperative HRV characteristics, patients were assigned to one of three phenotypical groups, specifically to HIGH, LOW or INTERMEDIATE heart rate variability. In order to differentiate among groups, the median cut-off of RMSSD of HRV, and of IIQTVI, were uniformly used. Patients with higher than median RMSSD, and lower (healthier) than median IIQTVI, were allocated to the HIGH variability group, whereas patients with lower than median RMSSD and higher than median IIQTVI were allocated to the LOW variability group. The remaining patients were grouped into INTERMEDIATE variability. In order to contrast higher versus lower HRV, the two groups HIGH and LOW were used for comparison.

**Figure 4:**
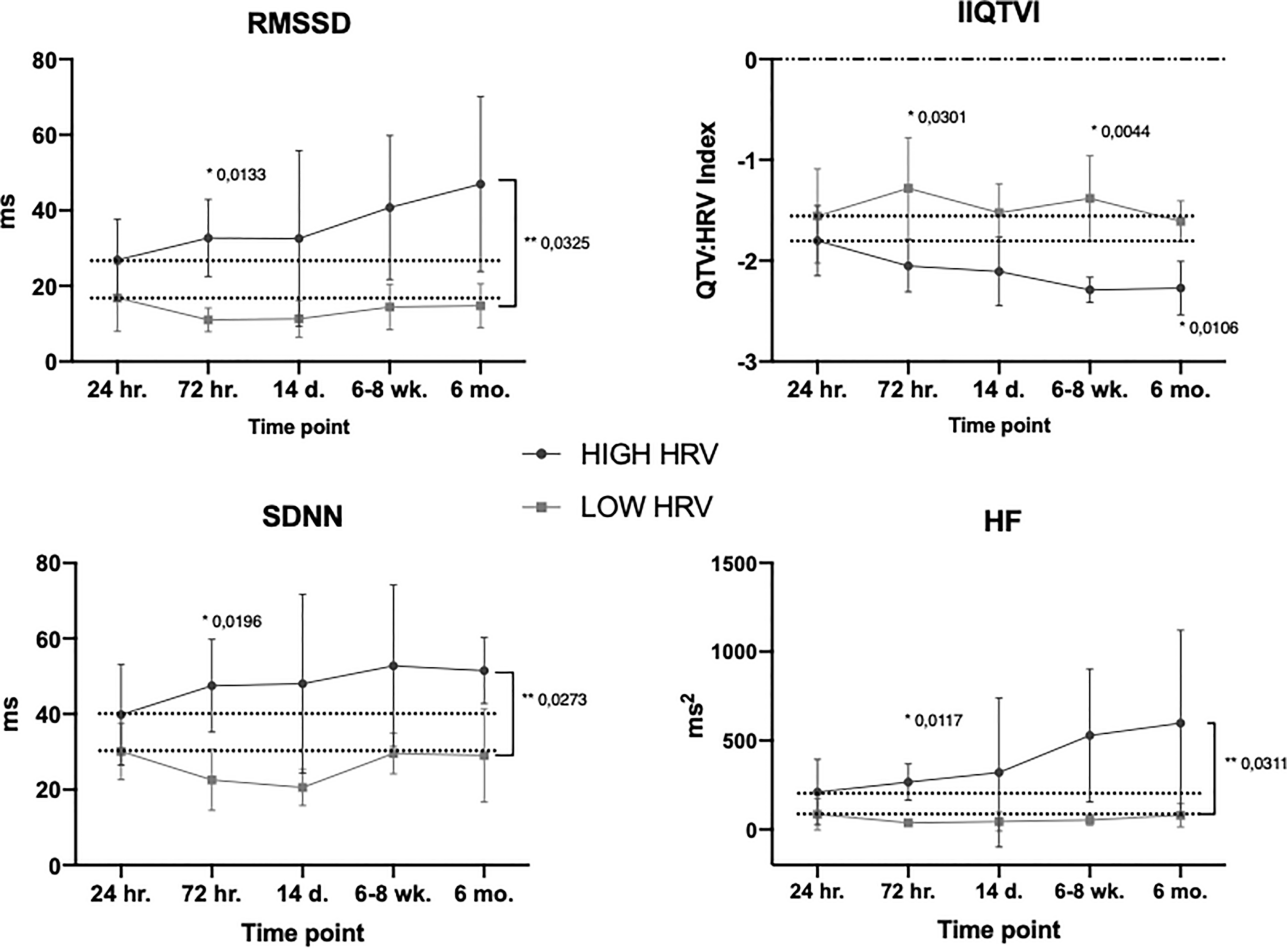
Temporal HRV subgroup trajectories. Mean values, SD. Dotted line is level at start of ‘response time’ (24hr.). * Bonferroni’s multiple comparison test, ** Group x time effect (mixed effect model). For the variable IIQTVI – the more negative the value, the stronger the variability. For all timepoints, see **Supplementary Figure 2**.

Comparing the temporal aspects of HRV with respect to patients’ pre-operative HRV-characteristics revealed distinct differences in response patterns. Patients in the HIGH group exhibited a more dynamic HRV response pattern to surgery over time whereas patients in the LOW group displayed an overall impaired response pattern to surgery throughout the perioperative period. Although with different baselines, the groups converged with lower variability after induction of anesthesia but followed by a steady increase starting 24 hours post-surgery for only the HIGH group (**Figure 4**).

#### 3.1.2 HRV Phenotypes and Molecular Inflammatory Response Patterns

**Figure 5:**
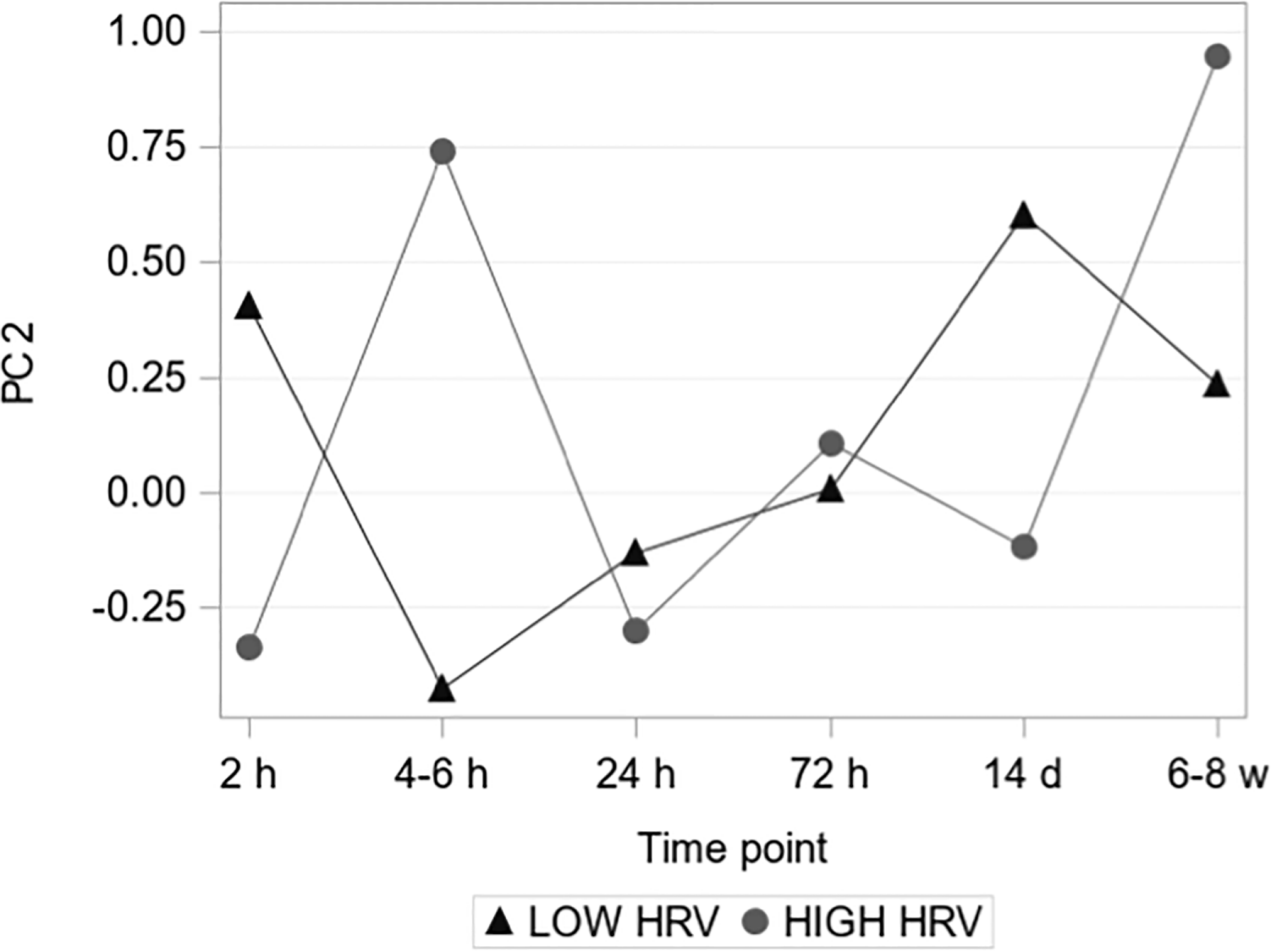
Principal component 2. The second PC showed a significant group-time interaction assessed by a mixed-effects model (P = 0.013 < 0.05/3 = 0.017, i.e. after Bonferroni correction).

To explore the relationship between pre-operative vagal nerve activity pattern and temporal systemic inflammatory response, a principal component analysis (PCA) was conducted. Based on their high fraction of measurements above baseline 77/92 serum biomarkers were included in this analysis. The PCA was performed on pre-operative values and the three first principal components (PC1-PC3) were identified, explaining 44% of the pre-operative biomarker variation. When the obtained principal components were applied to the postoperative standardized marker measurements, PC2 showed significant group-time interaction for HIGH versus LOW HRV-groups (**Figure 5**). The relative contribution of individual biomarkers to the separation of the inflammatory trajectories in PC2 was further explored among the 36 markers with loadings >0.1 (see **Supplementary Table 1**, for included markers). Defined molecular drivers underlying the earlier separation of molecular patterns were TGF-α and S100A12/EN-RAGE, and the later separation was characterized by CX3CL1, MMP1, NT-3, CXCL6 and FGF-21, all with nominal significance (see **Supplementary Figure 4** for individual marker trajectories).

### 3.2 *Ex Vivo* LPS Stimulation & Systemic Inflammatory Markers

**Figure 6:**
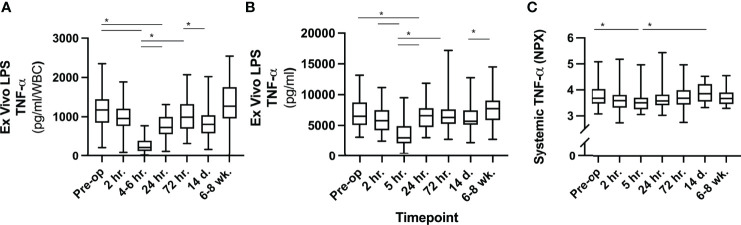
Analyses of TNF-α. **(A)** TNF-α release *ex vivo* following LPS stimulation divided by systemic WBC at same timepoint. **(B)** TNF-α release *ex vivo* following LPS stimulation without adjustment for WBC. **(C)** Systemic TNF-α in circulation (serum) expressed as normalized units (NPX) on a log scale. * P-values ≤0.05 Tukey´s multiple comparison test, not all significant differences between timepoints are outlined. LPS, lipopolysaccharide; WBC, white blood cell count; NPX, normalized protein units.

**Figure 7:**
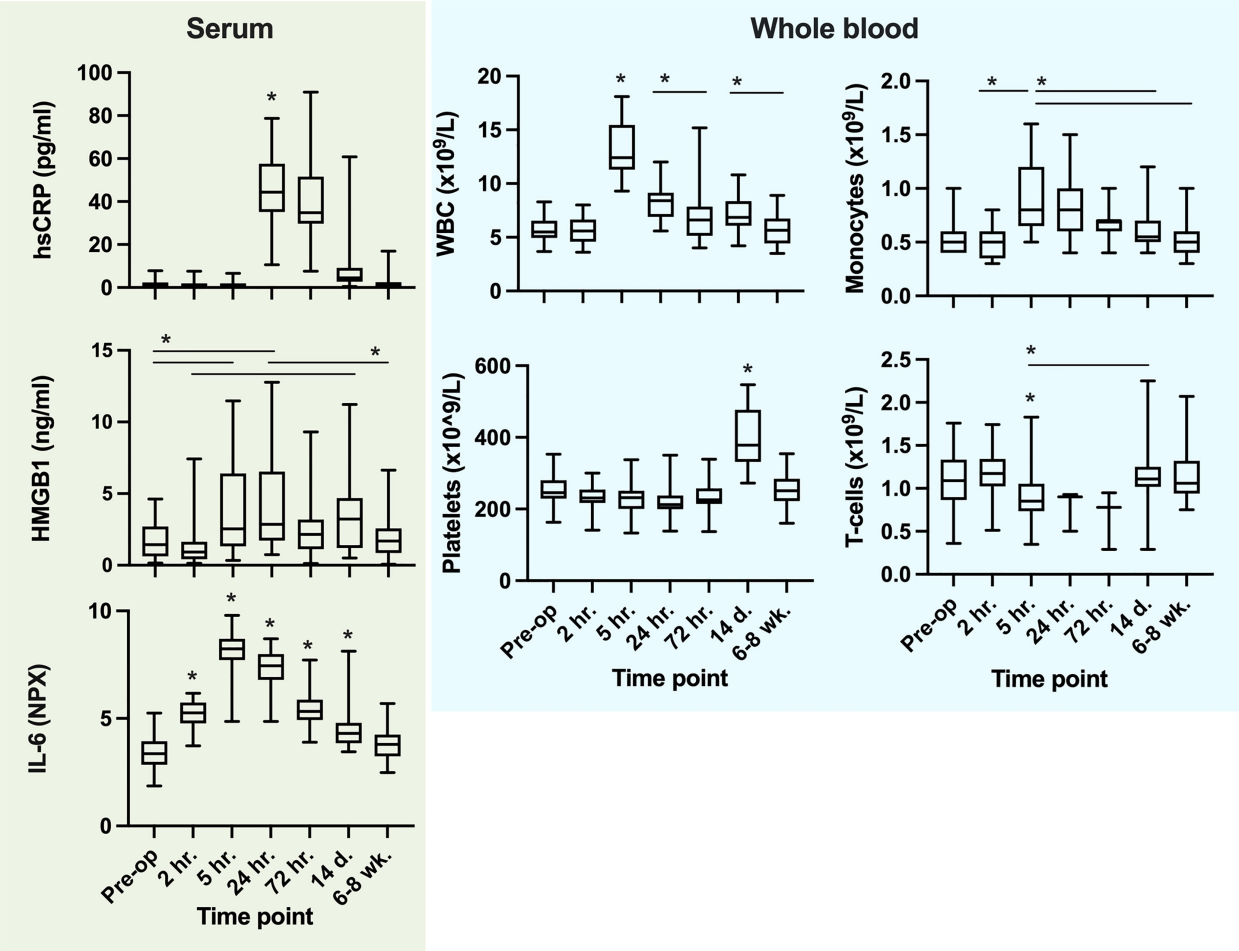
Systemic cytokines, alarmins (serum), cellular systemic trajectories and *ex vivo* LPS challenge-induced TNF-α release. For *Ex vivo* LPS challenge, TNF-α (pg/ml) is divided by WBC (x10^9) in order to describe the ‘reactivity per white blood cell’ to endotoxin. *P values ≤0.05 from the preceding timepoint or interval when present (Tukey´s multiple comparison test). Not all significant changes between timepoints are outlined.

There was a multiphasic response in *ex vivo* LPS-induced TNF-α release after surgery, with an initial pronounced depression (-79%) at 5 hours after skin incision followed by a secondary depression at 14 days, a pattern sustained both with and without adjustment for WBC (**Figure 6**). Systemic TNF-α levels, however, neither showed phasic reaction patterns nor dynamic changes over time. In parallel, there was a marked increase in WBC (+135%) and IL-6 (+135%), with peaks at 5 h post skin incision. hsCRP rose to a maximum (+2338%) at 24 hours after surgery. HMGB1 (non-stimulated serum) increased significantly at 5 h (+131%) and peaked at 24 hours after surgery (+158%), also with a biphasic temporal pattern. All patients had a significant rise in platelet counts at 14 days post-surgery (+57%). Systemic biomarkers of inflammation were normalized by 6-8 weeks post-surgery (**Figure 7**).

### 3.3 Monocytes, T-Cells & Platelets

The total number of monocytes (systemic CD14^+^ cells) after skin incision increased at 5 hours (+70%) and returned to baseline at 72 hours (**Figure 7**). Further subtype analysis revealed that the number of intermediate type monocytes (CD16^+^CD14^+^) was markedly depressed at 5 hours post-surgery, the non-classical type were significantly reduced, while the classical monocyte number increased in the circulation at the same time point (see **Supplementary Figure 3**, for subtype data). T-cell (CD3^+^) numbers were significantly reduced at 5 hours compared to 2 hours post skin incision. Platelet numbers rose significantly in all patients between 72 hours and 14 days and were normalized by 6-8 weeks after surgery (**Figure 7**).

Subgroup analysis revealed a difference in hsCRP resolution, where the HIGH HRV group had significantly lower values 72 hours post-surgery compared to the LOW HRV group (data not included). No other significant differences were evident between subgroups in inflammatory markers or cell numbers.

### 3.4 Cognitive Results

At 72 hours post-surgery five patients (20%) exhibited cognitive impairment (composite Z-score >1.0) and at 6-8 weeks two patients (8%) demonstrated the same impairment. In subtests, 7 patients (28%) at 72 hours and 9 patients (38%) at 6-8 weeks had a Z-score >2.0 in one of the seven derived variables. Four patients significantly improved their cognitive performance by 6-8 weeks post-surgery as compared to their preoperative results.

In the HIGH HRV group 2/6 individuals (33%) had a cognitive impairment at 72 hours, all with recovered performance by 6-8 weeks post-surgery. In the LOW HRV group, however, 4/7 patients (57%) had poor cognitive performance post-surgery, with two of these retaining cognitive impairment 6-8 weeks post-surgery and one fulfilling criteria for Post-op Neurocognitive Disorder (NCD). Considering poor performance on any individual ISPOCD subtest, 50% and 71% performed poorly (Z-score >2) in the HIGH and LOW HRV groups, respectively. However, these group differences were not statistically significant.

## 4 Discussion

In this study we performed an extensive temporal characterization of the human immune response to a standardized surgical procedure and corresponding associations with HRV. Profound changes in systemic inflammatory markers, immune cell counts and systemic immune reactivities occurred in the immediate postoperative period, with biphasic response patterns being observed. Furthermore, temporal recordings of HRV revealed distinct differences in HRV response patterns depending on the preexisting individual baseline HRV before surgery. Moreover, a PCA outlined that the pre-op HRV pattern associated with a differential molecular immune response pattern after surgery. HRV characteristics also exhibited a tendency to associate with cognitive outcomes, although the current study was insufficiently powered to yield statistically significant correlations.

The immune response to surgery is a robust, multilayered cascade of events that mirrors the responses to trauma or sepsis (35, 36). It can be argued that the characteristic surgery-induced inflammatory surge is a developmental reaction to microbial intrusion or tissue injury that triggers a purposeful change in behavior termed ‘sickness behavior’, that ultimately facilitates healing independent of the exact nature of infection or injury (5). There is growing evidence that this crucial response is partly regulated through complex neuro-immune reflex pathways. The CAP is a powerful reflex arc that participates in the regulation of peripheral inflammation (12, 37). In support of this peripheral immune regulation, we determined the novel finding of a close association between pre-operative HRV and postoperative inflammatory molecular response patterns.

HRV has been widely used to explore parasympathetic activity and the CAP in a range of clinical conditions of acute and chronic inflammation (18, 38–40). Moreover, baseline HRV has been suggested as a predictor of cardiovascular risk and all-cause mortality, especially in elderly cohorts (41, 42). Our finding that pre-operative HRV associates with differential inflammatory response patterns strongly suggests that HRV has the potential to identify patients at risk for adverse surgical outcomes. In a recent study of recovery from orthopedic surgery, Grote *et al*, reported that patients with a higher pre-operative HRV displayed a more rapid HRV-recovery and even enhancement of HRV post-surgery compared to patients with lower pre-op values (43). Similarly, our results indicate an increased variability during the weeks following surgery, but only in the HIGH HRV group. There is reason to believe that a healthy and dynamic HRV associates more with a timelier restoration of inflammatory homeostasis than does a lower and more static HRV (44). The exact connection between HRV and overall vagal tone, cardiac vagal tone or more localized vagal nerve activity is yet not fully elucidated (45).

The use of PCA in the exploration of HRV in relation to the immune response aims at unmasking subtle biological patterns that are not detectable when analyzing separate inflammatory markers due to the apparent risk of mass significance with the high number of explanatory variables in the data set. This PCA uncovered seven inflammatory biomarkers (TGF-α, S100A12/EN-RAGE, CX3CL1, MMP1, NT-3, CXCL6 and FGF-21) driving the HRV subgroup differences in the temporal immune response. As evident, these biomarkers represent regenerative, neurotrophic, and inflammatory pathways that warrant further in-depth exploration in larger patient samples to assess their individual relevance.

Recent studies reveal that the CAP relies on sympathetic nerves (i.e. the splenic nerve) as well as cholinergic neurons and targets more lymphatic tissues than the spleen (9–11). Not only the activity of the vagal nerve but also the balance of the ANS therefore needs to be considered when assessing neuroimmune regulation. However, as the balance of the sympathetic nervous system (SNS) and parasympathetic nervous system (PNS), previously assessed by LF : HF, is controversial, we mainly focused on the parasympathetic activity as the key driver of the CAP (46).

Consistent with the literature we recorded that levels of early systemic alarmins, such as HMGB1, increased within the first hours after skin incision. At the same time point we noted a marked reduction in white blood cell *ex vivo* release of TNF-α. This reduction in innate immune cell reactivity is likely to be associated with previously reported post-surgical immune depression (47). The cellular reponse to LPS is known to be reduced by opioids both *in vivo* and *ex vivo* (48, 49). In the current surgical population, opioid administration typically started during induction of anesthesia, i.e. before skin incision, and continued throughout surgery and in the immediate postoperative period, with oral opioids on prescription up to 10 days post surgery. Because the LPS response was well maintained during the most intense period of administration of anesthesia and analgetics (i.e. at 2 hours after skin incision) we argue that it is unlikely that the dynamic immune response is primarily dependent on an opioid effect.

We further demonstrate that the human response to surgery displays a simultaneous systemic biphasic molecular and cellular response pattern. The cellular response to surgery was evident by a rapid increase in systemic neutrophils and monocytes whereas the T-cell population apparently was affected at a later timepoint. These observations may fuel the concern of a transition of the acute innate immune activation due to surgery onto adaptive long-term alterations within the immune system (50). The observation of a delayed thrombocytosis at 2 weeks postsurgery support these concerns (51). Finally, we cannot exclude that anesthesia per se have temporary and shortlasting influences on the regulation of immune activity, as indicated by the abrupt changes in HRV following induction and withdrawal of anesthesia (52).

Because the exclusion criteria applied in this study were used to avoid known interference with HRV data, the study population mostly represents healthy individuals. In addition, this study was not sufficiently powered to assess cognitive outcomes in relation to HRV subgroups. Nonetheless, we did observe cognitive deterioration in several patients, and in one this was prolonged, indicating the relevance of this association. We are aware of potential hidden confounders in the ECG-based analysis of HRV such as frequent ectopy, pathological non-respiratory sinus or other arrhythmias or subclinical cardiac disease. To minimize confounders, such ECG abnormalities were identified by a clinical physiology ECG specialist and related results excluded if they interfered with accurate HRV analyses. Moreover, the long-term postoperative follow-up period may include medical events such as renewed surgical procedures or systemic infection that impact the immune system. As we chose to only include male patients in order to reduce surgical heterogeneity, corresponding studies with females are required.

Bridging gaps of knowledge regarding the human immune responses to surgical trauma can help us identify mechanisms that may prevent adverse postoperative brain outcomes. Improved understanding of autonomic nervous system (ANS)-dependent regulatory control over mechanisms of inflammation may provide clinically relevant tools for screening of at-risk patients, as well as for immune-modulating therapeutics.

## 5 Conclusions

This study contributes to the understanding of surgery-induced inflammatory responses and the potential role of the brain in regulation of systemic immune response to surgery. We uncovered a differential inflammatory response pattern closely linked to pre-existing HRV dynamics, with a potential impact on post-operative outcomes that warrants further clinical consideration and investigation.

## Data Availability Statement

The raw data supporting the conclusions of this article will be made available by the authors, without undue reservation.

## Ethics Statement

The studies involving human participants were reviewed and approved by Stockholm Regional Ethical Review board (2016/1745-31/1, 2021/00488). The patients/participants provided their written informed consent to participate in this study.

## Author Contributions

MH and JK planned the study, performed the research and wrote the manuscript. AS, AG and LK performed the research. JH, HH and RH planned the study and performed experiments. TS and AE planned the study and performed analyses. HZ performed experiments. FG contributed with senior statistical competence. LE planned the study, wrote the manuscript and supervised the work. All authors contributed to the article and approved the submitted version.

## Funding

This work has been supported by the Research Council Medicine Sweden [grant number 2020-01485], the Region Stockholm ALF grant, Sweden [#20200033] and the Brain Foundation, Sweden [#FO2021-0066]. HZ is a Wallenberg Scholar supported by grants from the Swedish Research Council [#2018-02532], the European Research Council [#681712], Swedish State Support for Clinical Research [#ALFGBG-720931], the Alzheimer Drug Discovery Foundation (ADDF), USA [#201809-2016862], the AD Strategic Fund and the Alzheimer’s Association [#ADSF-21-831376-C, #ADSF-21-831381-C and #ADSF-21-831377-C], the Olav Thon Foundation, the Erling-Persson Family Foundation, Stiftelsen för Gamla Tjänarinnor, Hjärnfonden, Sweden [#FO2019-0228], the European Union’s Horizon 2020 research and innovation programme under the Marie Skłodowska-Curie grant agreement No 860197 (MIRIADE), European Union Joint Program for Neurodegenerative Disorders (JPND2021-00694), and the UK Dementia Research Institute at UCL.

## Conflict of Interest

HZ has served at scientific advisory boards and/or as a consultant for Abbvie, Alector, Annexon, Artery Therapeutics, AZTherapies, CogRx, Denali, Eisai, Nervgen, Pinteon Therapeutics, Red Abbey Labs, Passage Bio, Roche, Samumed, Siemens Healthineers, Triplet Therapeutics, and Wave, has given lectures in symposia sponsored by Cellectricon, Fujirebio, Alzecure, Biogen, and Roche, and is a co-founder of Brain Biomarker Solutions in Gothenburg AB (BBS), which is a part of the GU Ventures Incubator Program (outside submitted work). TS, affiliated with Karolinska Institutet, conducts HRV analysis through his company Nicollier-Schlegel SARL, Switzerland.

The remaining authors declare that the research was conducted in the absence of any commercial or financial relationships that could be construed as a potential conflict of interest.

## Publisher’s Note

All claims expressed in this article are solely those of the authors and do not necessarily represent those of their affiliated organizations, or those of the publisher, the editors and the reviewers. Any product that may be evaluated in this article, or claim that may be made by its manufacturer, is not guaranteed or endorsed by the publisher.

## Supplementary Material

Supplementary Table 1 Inflammatory markers (serum) explored for group differences in PCA.
