# Supplementary material for: The Neuroimmune Response to Surgery – An Exploratory Study of Trauma-Induced Changes in Innate Immunity and Heart Rate Variability
Source: Front Immunol. 2022 Jul 7;13:911744. doi: 10.3389/fimmu.2022.911744 (PMC9301672; doi:10.3389/fimmu.2022.911744)
Supplement: Supplementary Table 1 — Inflammatory markers (serum) explored for group differences in PCA. [file Table_1.docx]

| **Size order** | **Marker** | **PC2 loadings** | **Explored** |
| --- | --- | --- | --- |
| 1 | LIF_R | 0.25655 | Yes |
| 2 | CCL25 | 0.22114 | Yes |
| 3 | FGF_5 | 0.21086 | Yes |
| 4 | CCL20 | -0.21020 | Yes |
| 5 | TRANCE | -0.19963 | Yes |
| 6 | OSM | -0.19092 | Yes |
| 7 | EN_RAGE | -0.18817 | Yes |
| 8 | SLAMF1 | 0.18145 | Yes |
| 9 | FGF_21 | -0.17686 | Yes |
| 10 | uPA | 0.16882 | Yes |
| 11 | CX3CL1 | 0.16683 | Yes |
| 12 | CCL23 | 0.16612 | Yes |
| 13 | CD5 | 0.15601 | Yes |
| 14 | TGF_alpha | -0.15165 | Yes |
| 15 | IL_17A | -0.15011 | Yes |
| 16 | IL6 | -0.14982 | Yes |
| 17 | TNFRSF9 | 0.14840 | Yes |
| 18 | SCF | 0.14752 | Yes |
| 19 | IL18 | -0.13971 | Yes |
| 20 | CD244 | 0.13713 | Yes |
| 21 | IL_18R1 | -0.13669 | Yes |
| 22 | CXCL1 | -0.13018 | Yes |
| 23 | SIRT2 | -0.12999 | Yes |
| 24 | ADA | -0.11971 | Yes |
| 25 | FGF_23 | 0.11910 | Yes |
| 26 | OPG | 0.11775 | Yes |
| 27 | Flt3L | 0.11582 | Yes |
| 28 | MCP_1 | -0.11486 | Yes |
| 29 | _4E_BP1 | -0.11408 | Yes |
| 30 | CXCL6 | -0.11233 | Yes |
| 31 | TWEAK | 0.11057 | Yes |
| 32 | MMP_1 | 0.11014 | Yes |
| 33 | DNER | 0.10739 | Yes |
| 34 | AXIN1 | -0.10680 | Yes |
| 35 | TNFSF14 | -0.10326 | Yes |
| 36 | NT_3 | 0.10014 | Yes |
| 37 | IL_15RA | 0.09819 | No |
| 38 | MCP_2 | 0.09266 | No |
| 39 | IFN_gamma | -0.09123 | No |
| **Supplementary Table 1. Inflammatory markers in PCA.** Explored are markers with a loading >0.1 in PC2. PCA, Principal Component Analysis; PC2, Principal Component 2. | | | |
|  |  |  |  |
| 40 | IL_10RA | 0.09074 | No |
| 41 | TNFB | 0.08737 | No |
| 42 | LAP_TGF_beta_1 | 0.08362 | No |
| 43 | CD6 | 0.08329 | No |
| 44 | IL_10RB | 0.08228 | No |
| 45 | STAMBP | -0.08126 | No |
| 46 | CCL28 | 0.08120 | No |
| 47 | GDNF | 0.08018 | No |
| 48 | HGF | -0.07739 | No |
| 49 | CDCP1 | 0.07663 | No |
| 50 | ST1A1 | -0.07401 | No |
| 51 | CCL3 | -0.07383 | No |
| 52 | CD8A | -0.07212 | No |
| 53 | CCL19 | -0.06863 | No |
| 54 | IL8 | 0.06702 | No |
| 55 | TRAIL | -0.06461 | No |
| 56 | IL7 | 0.05716 | No |
| 57 | MCP_3 | -0.04931 | No |
| 58 | Beta_NGF | 0.04922 | No |
| 59 | CXCL10 | -0.04838 | No |
| 60 | VEGFA | 0.04464 | No |
| 61 | MMP_10 | 0.04447 | No |
| 62 | CSF_1 | 0.04318 | No |
| 63 | CD40 | 0.03846 | No |
| 64 | IL_20RA | 0.03599 | No |
| 65 | MCP_4 | 0.03482 | No |
| 66 | PD_L1 | 0.02558 | No |
| 67 | CASP_8 | 0.02063 | No |
| 68 | CXCL11 | -0.02004 | No |
| 69 | FGF_19 | -0.01879 | No |
| 70 | CST5 | 0.01692 | No |
| 71 | IL10 | -0.01554 | No |
| 72 | CXCL5 | -0.01483 | No |
| 73 | CCL4 | -0.01013 | No |
| 74 | CXCL9 | -0.00978 | No |
| 75 | IL_12B | -0.00698 | No |
| 76 | CCL11 | 0.00274 | No |
| 77 | TNF | 0.00218 | No |
