# Supplementary material for: The Neuroimmune Response to Surgery – An Exploratory Study of Trauma-Induced Changes in Innate Immunity and Heart Rate Variability
Source: Front Immunol. 2022 Jul 7;13:911744. doi: 10.3389/fimmu.2022.911744 (PMC9301672; doi:10.3389/fimmu.2022.911744)
Supplement: Supplementary file 2 [file DataSheet_1.pdf]

Pearson Correlation Coefficients<br>Prob > |r| under H0: Rho=0<br>Number of Observations							
	rMSSD	SD1	HFLomb	IIQTVI	LF_Lomb	LF_HF	SDNN
rMSSD	1.00000<br><br>22	0.99714<br><.0001<br>22	0.91573<br><.0001<br>22	-0.48028<br>0.0237<br>22	0.86142<br><.0001<br>22	-0.20374<br>0.3631<br>22	0.95732<br><.0001<br>22
SD1	0.99714<br><.0001<br>22	1.00000<br><br>22	0.93427<br><.0001<br>22	-0.49643<br>0.0188<br>22	0.88493<br><.0001<br>22	-0.19079<br>0.3950<br>22	0.95493<br><.0001<br>22
HFLomb	0.91573<br><.0001<br>22	0.93427<br><.0001<br>22	1.00000<br><br>22	-0.46112<br>0.0308<br>22	0.92308<br><.0001<br>22	-0.26251<br>0.2379<br>22	0.86231<br><.0001<br>22
IIQTVI	-0.48028<br>0.0237<br>22	-0.49643<br>0.0188<br>22	-0.46112<br>0.0308<br>22	1.00000<br><br>25	-0.37591<br>0.0847<br>22	0.13323<br>0.5545<br>22	-0.58395<br>0.0022<br>25
LF_Lomb	0.86142<br><.0001<br>22	0.88493<br><.0001<br>22	0.92308<br><.0001<br>22	-0.37591<br>0.0847<br>22	1.00000<br><br>22	0.08800<br>0.6970<br>22	0.86784<br><.0001<br>22
LF_HF	-0.20374<br>0.3631<br>22	-0.19079<br>0.3950<br>22	-0.26251<br>0.2379<br>22	0.13323<br>0.5545<br>22	0.08800<br>0.6970<br>22	1.00000<br><br>22	-0.06879<br>0.7610<br>22
SDNN	0.95732<br><.0001<br>22	0.95493<br><.0001<br>22	0.86231<br><.0001<br>22	-0.58395<br>0.0022<br>25	0.86784<br><.0001<br>22	-0.06879<br>0.7610<br>22	1.00000<br><br>25

Supplementary Figure 1. Correlation matrix of HRV variables . Preoperative values.
SDNN, standard deviation of normal-normal heart beats; rMSSD, root mean square of successive differences; LF Lomb, low frequency by Lomb periodogram; HF, high frequency by Lomb; SD1, Standard deviation of Poincaréplot perpendicular to line of identity (instantaneous variability); IIQTVI, QT Variability Index from ECG lead II.
