# Supplementary material for: The Neuroimmune Response to Surgery – An Exploratory Study of Trauma-Induced Changes in Innate Immunity and Heart Rate Variability
Source: Front Immunol. 2022 Jul 7;13:911744. doi: 10.3389/fimmu.2022.911744 (PMC9301672; doi:10.3389/fimmu.2022.911744)
Supplement: Supplementary file 3 [file DataSheet_2.pdf]

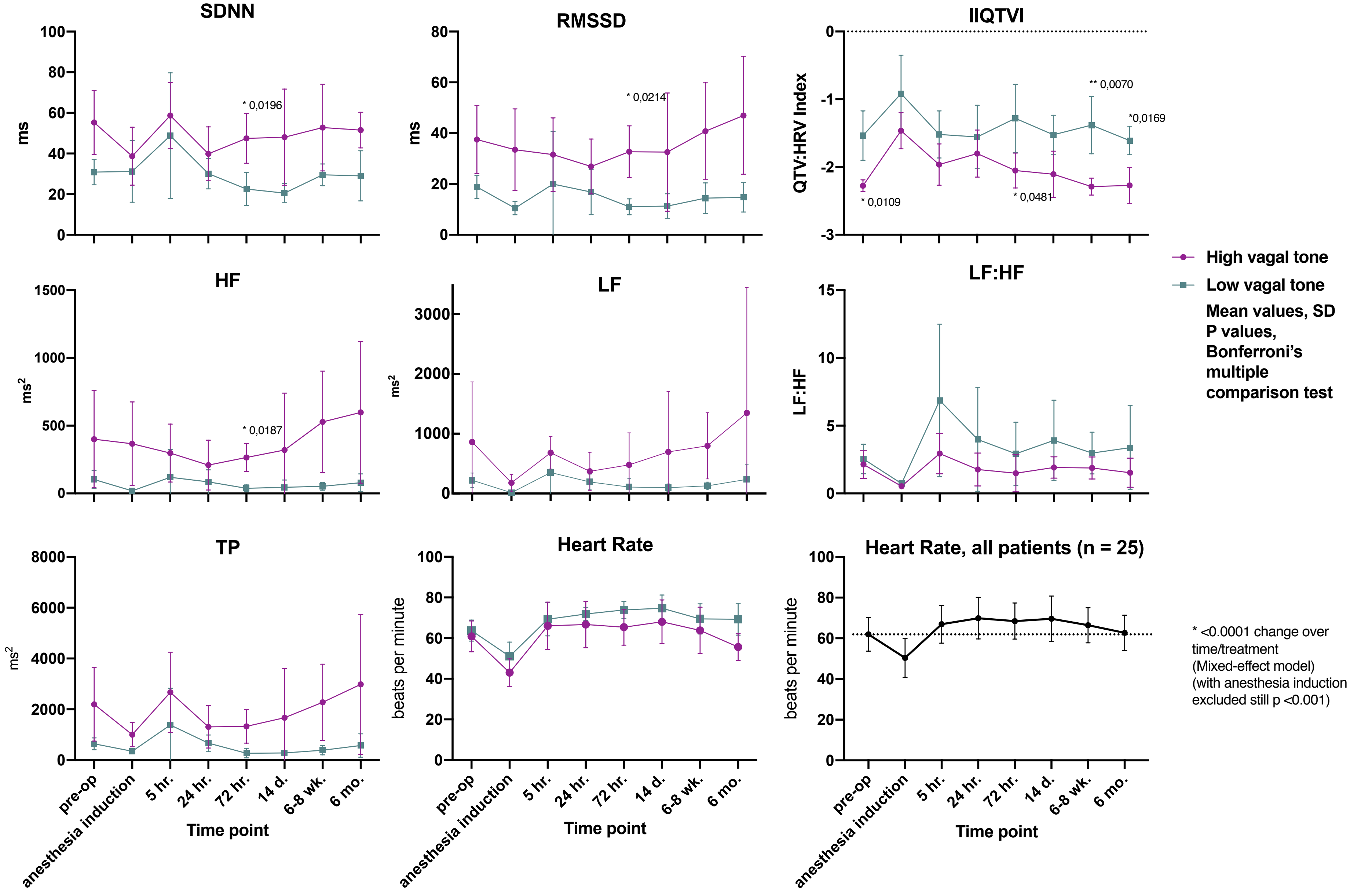

Supplementary Figure 2. Temporal HRV trajectories.

SDNN, standard deviation of normal-normal heart beats; rMSSD, root mean square of successive differences; LF Lomb, low frequency by Lomb periodogram; HF, high frequency by Lomb; TP, Total Power; IIQTVI, QT Variability Index
