# Supplementary figures and images for: The Neuroimmune Response to Surgery – An Exploratory Study of Trauma-Induced Changes in Innate Immunity and Heart Rate Variability
Source: Front Immunol. 2022 Jul 7;13:911744. doi: 10.3389/fimmu.2022.911744 (PMC9301672; doi:10.3389/fimmu.2022.911744)

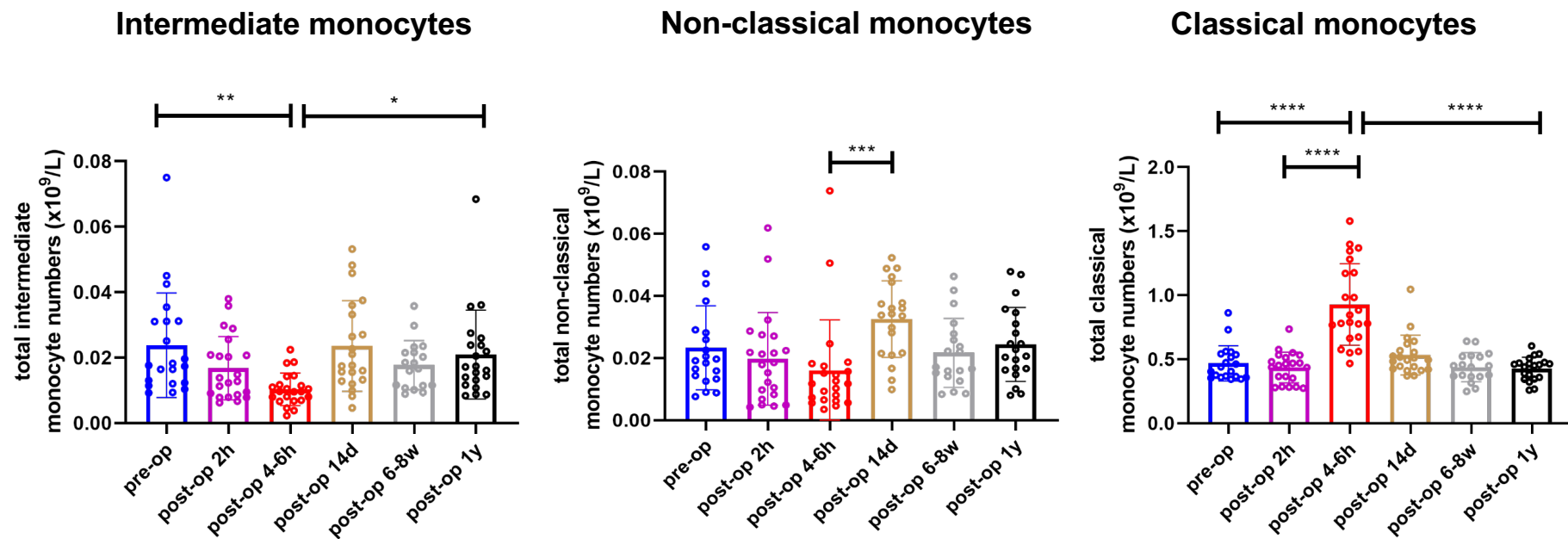

Supplementary Figure 3. Monocyte subtype data.

Supplement: Supplementary file 4 [file DataSheet_3.pdf]
