# Supplementary material for: The Neuroimmune Response to Surgery – An Exploratory Study of Trauma-Induced Changes in Innate Immunity and Heart Rate Variability
Source: Front Immunol. 2022 Jul 7;13:911744. doi: 10.3389/fimmu.2022.911744 (PMC9301672; doi:10.3389/fimmu.2022.911744)
Supplement: Supplementary file 5 [file DataSheet_4.pdf]

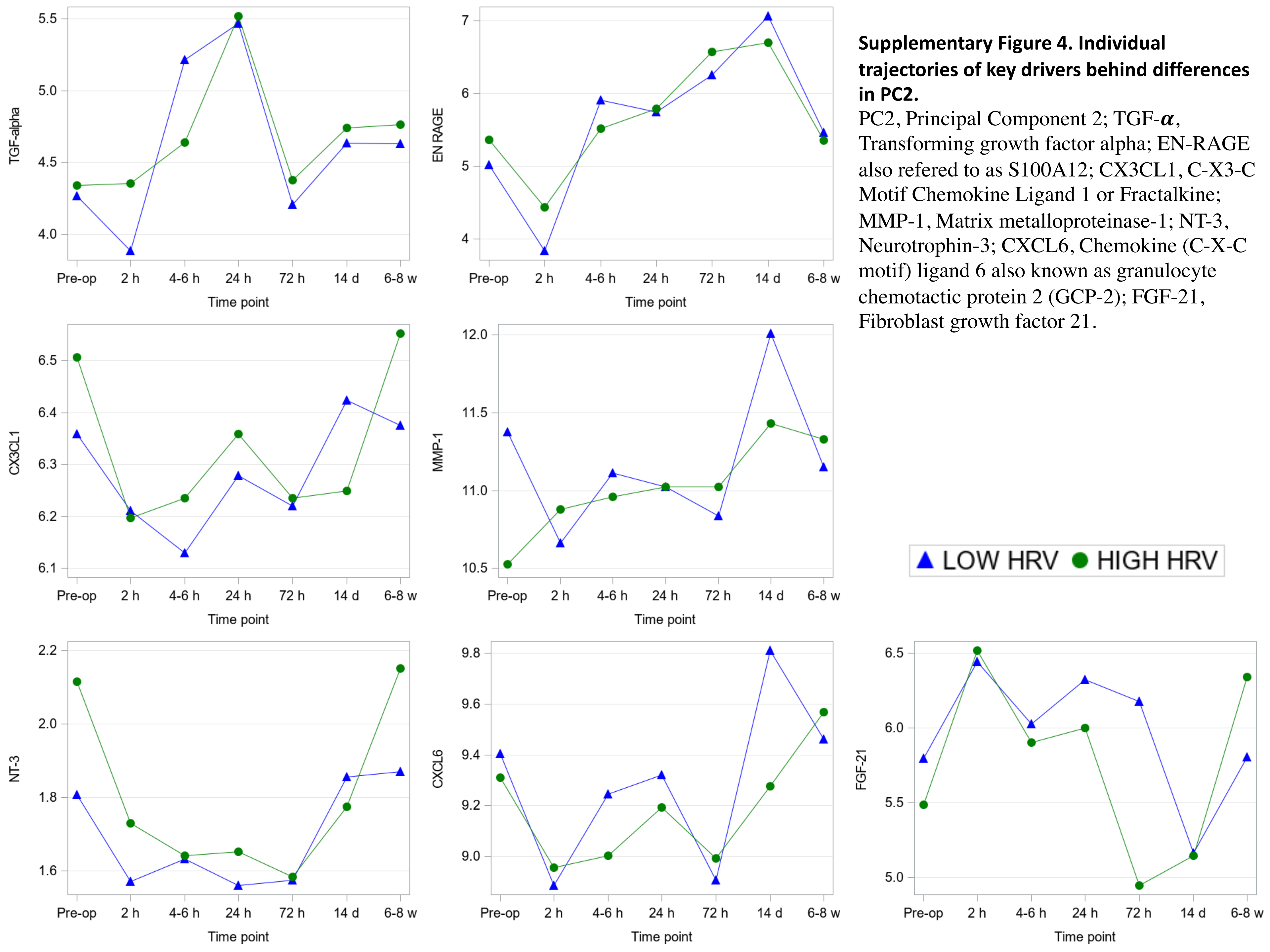

Supplementary Figure 4. Individual trajectories of key drivers behind differences in PC2.
PC2, Principal Component 2; TGF-α, Transforming growth factor alpha; EN-RAGE also refered to as S100A12; CX3CL1, C-X3-C Motif Chemokine Ligand 1 or Fractalkine; MMP-1, Matrix metalloproteinase-1; NT-3, Neurotrophin-3; CXCL6, Chemokine (C-X-C motif) ligand 6 also known as granulocyte chemotactic protein 2 (GCP-2); FGF-21, Fibroblast growth factor 21.
